# Biosynthetic Pathways and the Role of the Mas Receptor in the Effects of Angiotensin-(1–7) in Smooth Muscles

**DOI:** 10.1155/2012/121740

**Published:** 2011-11-17

**Authors:** Marcio Augusto Fressatto de Godoy, Larissa Pernomian, Ana Maria de Oliveira, Satish Rattan

**Affiliations:** ^1^Division of Gastroenterology & Hepatology, Department of Medicine, Jefferson Medical College, Thomas Jefferson University, Philadelphia, PA, USA; ^2^Laboratory of Pharmacology, Department of Pharmacology, Faculty of Medicine from Ribeirão Preto, University of São Paulo (USP), Avenida do Café s/n, 14040-903 Ribeirão Preto, SP, Brazil; ^3^Laboratory of Pharmacology, Department of Physics and Chemistry, Faculty of Pharmaceutical Sciences from Ribeirão Preto, University of São Paulo (USP), Avenida do Café s/n, 14040-903 Ribeirão Preto, SP, Brazil

## Abstract

Ang-(1–7) is produced via degradation of Ang II by the human angiotensin converting enzyme, also known as ACE2. In the cardiovascular system, Ang-(1–7) has been shown to produce effects that are opposite to those of Ang II. These include smooth muscle relaxation and cardioprotection. While the roles of Ang-(1–7) in other systems are currently topic of intense research, functional data suggest a relaxation action in gastrointestinal smooth muscles in a way that corroborates the results obtained from vascular tissues. However, more studies are necessary to determine a relevant role for Ang-(1–7) in the gastrointestinal system. The Ang-(1–7) actions are mediated by a distinct, functional, Ang-(1–7) receptor: the *Mas receptor* as shown by diverse studies involving site-specific binding techniques, selective antagonists, and targeted gene deletion. This paper provides an overview of the functional role and the molecular pathways involved in the biosynthesis and activity of Ang-(1–7) in diverse systems.

## 1. A Brief Historical Overview

Until the late 80s, it was thought that most of the biological activity of angiotensin peptides was based on their interaction with the AT_1_ receptor at the C-terminal side chain of a phenylalanine residue in the position 8 (Phe^8^) [1, 2]. Consequently, it was assumed that fragments of Ang II lacking the C-terminal Phe^8^ were biologically inactive [3]. A number of studies have shown that the N-terminal heptapeptide angiotensin-(1–7) [Ang-(1–7)], also named as des-[Phe^8^]-angiotensin II [4], lacked vasopressor effect [5, 6], aldosterone release activity [5], and central dipsogenic action [7].

However, in 1988, Santos and coworkers showed that Ang-(1–7) was produced as the main metabolite of angiotensin I (Ang I) in dog brainstem and spinal cord, which is produced even in the presence of angiotensin-converting enzyme (ACE) inhibitors, suggesting an ACE-independent route [8]. Further studies have shown that Ang-(1–7) stimulates arginine vasopressine (AVP) release from the rat hypothalamo-neurohypophysial system (HNS) with potency comparable to angiotensin II (Ang II) [9]. These findings triggered general scientific interest in the area with a series of studies involving site-specific, functional antagonism, and targeted-gene deletion that, among other techniques, have resulted in the identification of ACE2 and of the *Mas* receptor as the main agents responsible for the biosynthesis and actions of Ang-(1–7) at the molecular level. 

This paper provides an overview of the molecular pathways involved in the biosynthesis and activity of Ang-(1–7) in the vascular and gastrointestinal systems with emphasis on their smooth muscle structures, and the limited availability of such information in the gastrointestinal tract.

## 2. Biosynthesis and Degradation of Ang-(1–7)

ACE2 is the enzyme responsible for the biosynthesis of Ang-(1–7). ACE2 is a membrane-associated zinc metalloprotease and a homologue of the human ACE isoforms, which is highly expressed in several tissues such as human heart, kidney, lungs, and testis [10, 11]. There is also evidence for the presence of ACE2 in smooth muscles: in the cardiovascular system, studies have shown the expression of ACE2 in the media of thoracic aorta and common carotid arteries from spontaneously hypertensive rats (SHRs) [12]. In line with these studies, ACE2 has also been shown to be expressed in vascular smooth muscle cells (VSMCs) isolated from rat aorta [13, 14], and in renal and mesenteric arteries from spontaneously hypertensive stroke-prone rats (SHRSP) [15]. In the gastrointestinal system, studies suggest a role for local biosynthesis of Ang-(1–7). These include an inhibitory effect in the basal tone of the internal anal sphincter [16] and the identification of ACE2 mRNA in the stomach wall of rats [17]. In addition, studies using quantitative real-time polymerase chain reaction (QRT-PCR) have made the novel observation that ACE 2 shows levels of expression in the gastrointestinal system that are comparable to those in the cardiovascular system. Particularly high levels of ACE2 have been found in duodenum, jejunum, ileum, caecum, and colon. Therefore, consideration should also be given to a potential role for ACE2 in gastrointestinal physiology and pathophysiology [18]. However, other than this, there is relatively scarce information about formation and actions of Ang-(1–7) in the gastrointestinal system.

Despite its homology with ACE, ACE2 is functionally different as it acts as a C-terminal carboxypeptidase rather than a C-terminal dipeptidase by cleaving the C-terminal of a residue of Leu in the structure of Ang I or a residue of Phe in Ang II. This activity, respectively, generates angiotensin-(1–9) [Ang-(1–9)] [10, 11] or Ang-(1–7) [11, 19]. 

There are also pharmacological differences between ACE and ACE2. ACE2 is insensitive to classic ACE inhibitors such as lisinopril [10, 11], enalaprilat, or captopril [11] as well as other ACE inhibitors [20]. The differential sensitivity to ACE inhibitors results from amino acid substitutions in the substrate binding site from ACE2. Studies show that ACE2 has an Arginine (Arg) residue in the position 273 instead of the Glutamine (Gln) residue normally found in ACE [21]. This substitution allosterically impairs the interaction between ACE2 and classic ACE inhibitors because of the larger size of Arg^273^, which limits the size of the S_2_′ substrate subsite in ACE2 [21]. 

As mentioned above, ACE2 catalyzes the conversion of both Ang I and Ang II into smaller fragments. Donoghue et al. [10] first showed that ACE2 is able to hydrolyze Ang I. Following this, Vickers et al. [19] showed that ACE2 more efficiently hydrolyzes Ang II than Ang I since the latter is only partially hydrolyzed. ACE2 and the resulting formation of Ang-(1–7) play an important role in the cardiovascular system. Studies by Igase et al. [12] have shown that blockade of AT_1_ receptors in the media layer of thoracic aortas isolated from SHR resulted in significant increases in the expression of ACE2 at the mRNA and protein levels. This effect was consistently associated with increased levels of Ang-(1–7), suggesting that ACE2 generates Ang-(1–7) locally. Corroborating these results, studies by Lavrentyev and Malik [14] have found that reduction in ACE2 expression is followed by a reduction in the cellular levels of Ang-(1–7) in rat aorta VSMC. Together, these studies suggest the involvement of ACE2 in the generation of Ang-(1–7) within the VSMC. Similar results have been found in human coronary vessels [22] and rat stomach wall [17]. 

In addition to ACE2, there are other enzymes that have been reported to release Ang-(1–7). Endopeptidases are the first example and have been implicated in the biosynthesis of Ang-(1–7) mainly from Ang I. As an example, prolyl endopeptidase converts Ang I into Ang-(1–7) as shown in NG108-15 neuroblastoma versus glioma hybrid cells [23] and in endothelial cells from human and bovine aorta as well as in umbilical veins [24]. As another example, neutral endopeptidase (NEP or neprilysin) accounts for the generation of most of the circulating Ang-(1–7) derived from Ang I as suggested by studies in SHR and Wistar-Kyoto (WK) rats [25]. In addition, thimet oligopeptidase (EC 3.4.24.15) forms Ang-(1–7) from Ang I in VSMC [26]. The catalytic action of these peptidases is responsible for the efficiency of the Ang I-dependent pathways in the biosynthesis of Ang-(1–7) because ACE2 hydrolyzes Ang II rather than Ang I [19, 20]. 

ACE has also been reported to be able to release Ang-(1–7). Even though it does not catalyze the generation of Ang-(1–7) from Ang I or Ang II, ACE is able to cleave Ang-(1–9) leading to the generation of Ang-(1–7) [20]. Furthermore, ACE is an important component in the catabolism of Ang-(1–7). 

The first evidence that pointed a potential role for ACE in the degradation of Ang-(1–7) came from the findings obtained by Kohara et al. [27]. In this study, the authors observed that the chronic treatment of SHR or WK rats with the ACE inhibitors ceranopril or lisinopril augmented the circulating levels of Ang-(1–7). Corroborating with Kohara et al. [27], Luque et al. [28] showed that the chronic therapy of essential hypertensive subjects with captopril reduced the diastolic blood pressure without changing the plasma levels of Ang II while increasing the levels of Ang-(1–7). Taken together, these findings suggest a potential role for ACE in the degradation of Ang-(1–7), which may be involved in the antihypertensive effects from ACE inhibitors. 

Indeed, *in vitro *experiments obtained by Chappell et al. [29] showed that ACE cleaves the Isoleucine (Ile)^5^-His^6^ bond of Ang-(1–7) with a high specificity constant. The hydrolysis of Ang-(1–7) catalyzed by ACE involves the N-domain of the enzyme [30] and leads to the formation of angiotensin-(1–5) [Ang-(1–5)] [29]. 

## 3. Effects Elicited by Ang-(1–7)

Studies show that Ang-(1–7) has a cardioprotective role mainly attributable to counteraction of the Ang II effects, which contribute to maintaining the vascular homeostasis and attenuating the progression of atherogenesis [31, 32] among other effects as further explained below. 

Campagnole-Santos et al. [33] were the first to demonstrate the vascular effects induced by Ang-(1–7). The authors observed that the injection of Ang-(1–7) in the rat dorsal motor nucleus of the vagus elicited a centrally mediated hypotension similar to that induced by Ang II. Later, the authors showed that intracerebroventricular injections of Ang-(1–7) increased the baroreflex sensitivity for the control of the heart rate in conscious rats, suggesting that Ang-(1–7) facilitates the baroreflex by inducing depressor effects into the dorsal medulla [34]. In this context, Benter et al. [35] found that systemic injections of Ang-(1–7) transiently reduced the systolic pressure in SHR, suggesting a potential counterregulatory effect of Ang-(1–7) on the pressor effects elicited by the main agents that increase the total peripheral resistance during hypertension, like Ang II and *α*_1_-adrenergic agonists. Later, the authors confirmed their hypothesis [36] by showing that the intravenous administration of Ang-(1–7) reduced the pressor responsiveness to the *α*_1_-adrenergic agonist phenylephrine, and to Ang II, and improved the sensitivity of the reflex control of the heart rate in SHR. These results were suggestive that Ang-(1–7) activated antihypertensive mechanisms.

Additional studies showed that Ang-(1–7) was responsible for the hypotensive effects of ACE inhibitors like lisinopril and losartan. Experiments using monoclonal antibodies selective for Ang-(1–7) showed that the antihypertensive effects of lisinopril and losartan were reversed by scavenging the circulating Ang-(1–7) in SHR [29, 30, 37, 38]. Following diverse studies showed that Ang-(1–7) has a relaxation effect of its own. Meng et al. showed that Ang-(1–7) caused a mild dilatation of cerebral arterioles via a mechanism that involves the release of cyclooxygenases (COX) metabolites [39]. Pörsti et al. [40] demonstrated that Ang-(1–7) induced an endothelium- and nitric oxide synthase (NOS) metabolites-mediated relaxation in porcine coronary arteries. Similar findings were obtained by Brosnihan et al. [41], who showed an endothelium- and NOS metabolites-mediated relaxation evoked by Ang-(1–7) in canine coronary artery. Comparable results were found in other animal species using a number of tissues suggesting that Ang-(1–7) has a wide-spread relaxation effect in the cardiovascular system via NO release [42–44]. In the vascular system, the counterregulatory actions of Ang-(1–7) over the effects produced by Ang II occur at the molecular level. Sampaio et al. [45] have shown that Ang-(1–7) inhibits the assembly and activation of NAD(P)H oxidase induced by Ang II by inhibiting Ang II-induced phosphorylation of p47phox, which is crucial to the NAD(P)H oxidase activation. This Ang II-mediated mechanism results in O_2_^−^ generation [46], NO inactivation [47], and has been correlated with diabetes. During diabetes mellitus, the vascular endothelium is an important source of NAD(P)H oxidase-derived O_2_^−^, which is involved in the reduction of endothelial NO availability and in the consequent endothelial dysfunction [47, 48]. Therefore, the counteractive effects by Ang-(1–7) over Ang II make it a potentially important therapeutic target to attenuate the endothelial dysfunction and in treating diabetes mellitus.

Ang-(1–7) also has antiproliferative effects in the cardiovascular system. Freeman et al. [49] have shown that Ang-(1–7) inhibits incorporation of mitogen-stimulated thymidine in rat aortic VSMC thus inhibiting cellular growth. The mechanism was characterized by Tallant and Clark [50], who showed that Ang-(1–7) attenuates the mitogenic activity of MAPK by a cAMP-dependent protein kinase (PKA), which is activated after a PGI_2_-mediated increase in cAMP production. In addition, Strawn et al. [51] have shown that Ang-(1–7) treatment reduced the DNA synthesis and the cross-sectional area of neointima in rat carotid artery injured by balloon catheter. In line with these results, Langeveld et al. [52] observed that Ang-(1–7) treatment reduces the neointimal thickness in rat abdominal aorta after stent implantation and restores the impaired endothelial function. Many other studies also support the concept that Ang-(1–7) plays an important role in neointimal re-endothelization and inhibition of neointimal formation and restenosis [52–54]. 

Studies also provide evidence for a role for Ang-(1–7) in reducing thrombus formations. Experiments in rat vena cava have shown that Ang-(1–7) reduces the thrombus weight and platelet adhesion to fibrilar collagen by a mechanism involving NOS and COX metabolites [55]. In addition, Tesanovic et al. [56] found that the chronic treatment with Ang-(1–7) reduced the development of atherosclerotic lesion in ApoE (−/−) and high-fat diet-fed mice, followed by an increase in the local expression of *e*NOS. These findings have opened new perspectives on the promising vasculo- and atheroprotective effects of Ang-(1–7) suggesting therapeutic potentials for the atherosclerosis, thrombosis, and atherothrombosis. 

## 4. *Mas* Receptor 

The *Mas* receptor was first cloned and sequenced by Young et al. [57] in cotransfected NIH 3T3 cells from nude mice and revealed a very hydrophobic protein, containing seven potential transmembrane domains. 

A connection between the *Mas* receptor and Ang-(1–7) was first established by Santos et al. [58]. Using binding studies performed in kidney sections from wild-type and *Mas*-deficient mice, the authors demonstrated that Ang-(1–7) binding was absent in kidneys from *Mas*-deficient mice, but preserved in wild-type membranes. In addition, the authors also showed that Ang-(1–7) binding was preserved in membranes isolated from AT_1_- or AT_2_-deficient animals, suggesting a twofold conclusion: (1) that Ang-(1–7) has limited interactions with AT_1_ and AT_2_ receptors and (2) that it mainly binds to *Mas* receptors [58]. These results were confirmed by further studies showing high affinity sites for Ang-(1–7) in *Mas* receptors. The results also showed that the *Mas* receptor has very low affinity for AT_1_ or AT_2_ ligands, excluding the hypothesis that Ang-(1–7) would directly interact with AT_1_ or AT_2_ receptors [58].

The first evidence for a functional role for the *Mas* receptor as the mediator of the Ang-(1–7) effects in the vascular system was also provided by Santos et al. [58]. The authors showed that the relaxation induced by Ang-(1–7) in mouse aorta was absent in *Mas*-deficient mice. The authors were also the first group to develop a selective antagonist for the *Mas* receptor called D-Ala^7^-Ang-(1–7) also known as A-779 [58, 59]. These findings fostered publication of many functional studies supporting a role for *Mas* receptors in the effects induced by Ang-(1–7). These include indirect (centrally mediated) antihypertensive effects [60] and local effects at the cellular [61] and molecular [44] levels. Tirapelli et al. [61] have shown that A-779 inhibited vasorelaxant effects caused by Ang-(1–7) in rat carotid artery in a concentration-dependent fashion. Sampaio et al. [44] demonstrated that A-779 blocks the phosphorylation of *e*NOS induced by Ang-(1–7) in human aortic endothelial cells, suggesting the involvement of *Mas* receptors in this effect. A-779 also blocked the inhibitory effect of Ang-(1–7) on VSMC growth, suggesting the participation of *Mas* receptors in the antiproliferative action of Ang-(1–7) [62]. 

There is also functional evidence directly supporting a role for *Mas* receptors in the antithrombotic effect of Ang-(1–7). A-779 dose-dependently inhibits the Ang-(1–7)-induced reduction in venous thrombus weight [55]. In addition, A-779 reduces the development of atherosclerotic lesion by Ang-(1–7) in mice. Interestingly, this effect was more evident when A-779 was combined with the AT_2_-antagonists, suggesting an important interaction between *Mas* and AT_2_ receptors in mediating the atheroprotective effect of Ang-(1–7) [56].

Multipronged studies also show a potential role for* Mas* receptors and Ang-(1–7) in the regulation of gastrointestinal smooth muscle motility. Studies by De Godoy et al. in Dr. Rattan's laboratory [16] have shown that Ang-(1–7) dose-dependently reduces the basal tone of spontaneously contracted internal anal sphincter of rats. The studies also show that this relaxation effect is abolished by A-779. In addition, the studies have shown that A-779 had no significant effect on the contractile response of Ang II [16]. Whether Ang-(1–7) has an important role in the gastrointestinal system remains to be determined.

In closing, Ang-(1–7) relaxes smooth muscles via interactions with the *Mas* receptor that elicit well-known molecular mechanisms of relaxation such as the release of NO. Limited studies suggest a potential role for Ang-(1–7) in regulating motility of gastrointestinal smooth muscles, but additional studies are necessary to further determine Ang-(1–7) actions and therapeutic potentials in the gastrointestinal system. On the other hand, the effects of Ang-(1–7) in the cardiovascular system are better understood and provide strong evidence for the cardiovascular protective action that can be selectively modulated via *Mas* ligands and have important therapeutic implications for human therapy.
